# Extracellular Vesicles Released from Cancer Cells Promote Tumorigenesis by Inducing Epithelial to Mesenchymal Transition via β-Catenin Signaling

**DOI:** 10.3390/ijms24043500

**Published:** 2023-02-09

**Authors:** Vamshikrishna Malyla, Keshav Raj Paudel, Gabriele De Rubis, Nicole G. Hansbro, Philip M. Hansbro, Kamal Dua

**Affiliations:** 1 Discipline of Pharmacy, Graduate School of Health, University of Technology Sydney, Sydney, NSW 2007, Australia; 2 Centre for Inflammation, Faculty of Science, School of Life Sciences, Centenary Institute and University of Technology Sydney, Sydney, NSW 2007, Australia; 3 Australian Research Centre in Complementary and Integrative Medicine, Faculty of Health, University of Technology Sydney, Ultimo, NSW 2007, Australia

**Keywords:** lung cancer, extracellular vesicles, tumorigenesis, β-catenin signaling, biomarkers, EMT

## Abstract

Lung cancer is the leading cause of cancer-related deaths globally, in part due to a lack of early diagnostic tools and effective pharmacological interventions. Extracellular vesicles (EVs) are lipid-based membrane-bound particles released from all living cells in both physiological and pathological states. To understand the effects of lung-cancer-derived EVs on healthy cells, we isolated and characterized EVs derived from A549 lung adenocarcinoma cells and transferred them to healthy human bronchial epithelial cells (16HBe14o). We found that A549-derived EVs carry oncogenic proteins involved in the pathway of epithelial to mesenchymal transition (EMT) that are regulated by β-catenin. The exposure of 16HBe14o cells to A549-derived EVs resulted in a significant increase in cell proliferation, migration, and invasion via upregulating EMT markers such as E-Cadherin, Snail, and Vimentin and cell adhesion molecules such as CEACAM-5, ICAM-1, and VCAM-1, with concomitant downregulation of EpCAM. Our study suggests a role for cancer-cell-derived EVs to induce tumorigenesis in adjacent healthy cells by promoting EMT via β-catenin signaling.

## 1. Introduction

EVs are lipid-based membrane-bound particles released from every living cells in both physiological as well as pathological states [1]. EVs carry numerous physiological biomolecules, such as DNA, RNA, lipids, and proteins, and therefore play a key role in cell-to-cell communication [1,2,3,4]. Based on the mechanism of biogenesis, size, characteristic surface markers such as CD63+/CD81+ and Annexin V, and method of collection, EVs are broadly divided into two subtypes: either small EVs (sEVs) or exosomes that are <100–200 nm, or medium to large EVs (m/lEVs) which are >200nm [1]. EVs have been extensively studied in the past couple of decades, and the importance of EVs lies primarily in the transport of physiological biomolecules from parent cells to recipient cells [5]. Cancer-cell-derived EVs can also play an important role in cell–cell communication, however, this remains incompletely defined, particularly in the context of tumorigenesis [6,7,8,9]. At the same time, there is a paucity of effective pre-clinical models to study disease pathophysiology which contributes to even now a poor understanding of the disease [10,11,12]. As a result, lung cancer has a very low five-year survival rate of around 15%, and an estimated 1.9 million cases are predicted to result in 0.6 million deaths in the United States alone in 2022 [13,14,15].

Cancer-cell-derived EVs (C-EVs) carry different biomarkers to their originating parent cells. As C-EVs are found in various biofluids, they can potentially serve as a diagnostic tool for cancer. For example, cancer markers such as CD91, CD317, and epidermal growth factor receptor (EGFR) are detected as membrane proteins on C-EVs, and a panel of 12 miRNAs was detected in EVs as RNA markers [6,7]. C-EVs also have the capacity to induce tumorigenesis in recipient cells by transferring oncogenic cargo; for example, mutant beta-catenin in EVs derived from colorectal cancer can activate WNT signaling in recipient cells [8]. Moreover, C-EVs are known to interact with different cell types such as endothelial cells and immune cells and are found in different organs such as the lymph nodes and bone marrow, wherein the capacity to induce tumorigenesis can have serious deleterious consequences for the individual [16,17,18,19]. Therefore, a better understanding of C-EVs not only helps in the detection of lung cancer via biomarker identification, but also helps in understanding tumorigenesis, and therefore may contribute to the design of effective therapeutic interventions.

Epithelial–mesenchymal transition (EMT) is a series of processes whereby epithelial cells lose their defining phenotype and gain mesenchymal characteristics, and which is a key driver of cancer metastasis [20]. Identification of EMT can be conducted by analyzing the changes in expression of well-characterized epithelial markers such as E-Cadherin and ß-catenin and mesenchymal markers such as Vimentin, Snail, and N-Cadherin [21]. These molecules are also significant because they can promote tumorigenesis by enhancing migration and proliferation, eventually leading to metastasis [22].

Until now, the effects of lung C-EVs on healthy recipient cells is not well characterized. To interrogate the role of lung C-EVs in tumorigenesis, and to evaluate their use as biomarkers for lung cancer, we isolated medium C-EVs released from lung adenocarcinoma cells (A549) into the culture supernatant and characterized them by three well-established methods: flow cytometry (for surface markers), Western blot (for protein markers), and diffraction light scattering (for size). Then, we profiled the A549 C-EVs by protein array to reveal novel markers and studied the potential of these lung C-EVs to induce tumorigenesis in healthy human bronchial epithelial cells.

## 2. Results

### 2.1. Isolation and Characterization of EVs 

**Figure 1:**
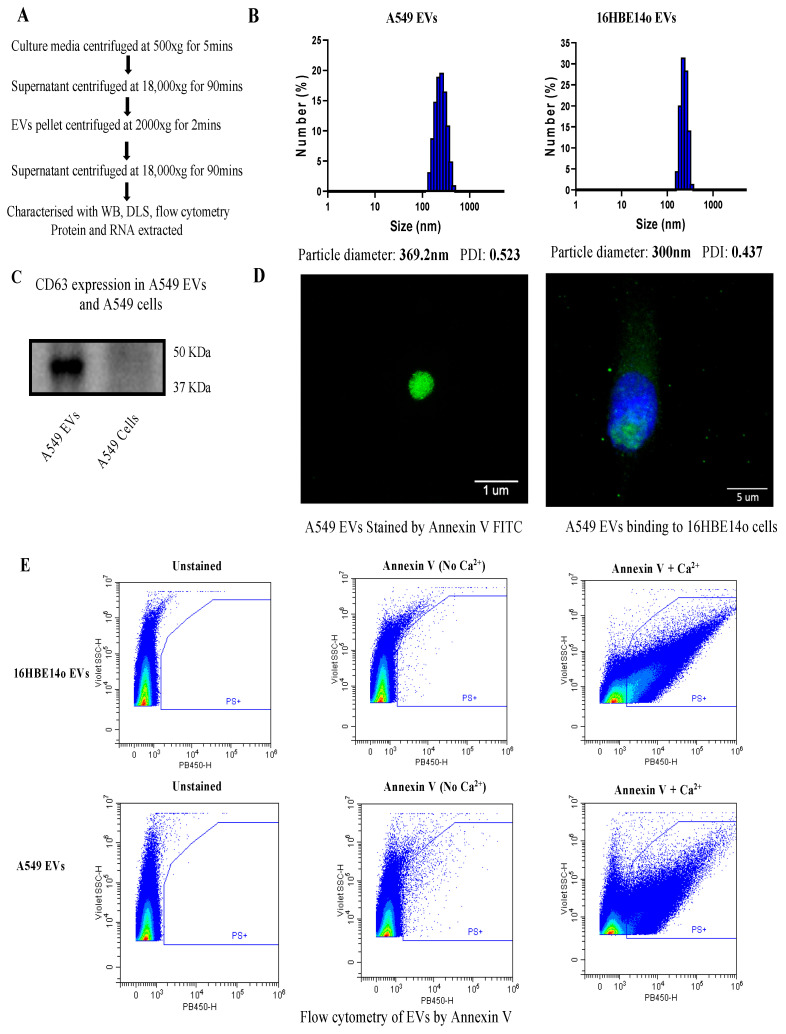
Isolation and characterization of EVs. (**A**) Flowchart showing the isolation of A549 and 16HBE14o EVs by high-speed centrifugation. (**B**) Size distribution of A549 and 16HBE14o EVs by DLS indicating both EVs are in the size range of medium EVs (>200–1000 nm). (**C**) Characterization of protein expression of tetraspanin marker CD63 on A549 EVs using Western blot. (**D**) Annexin V-FITC staining of EVs. Green fluorescence signal indicates positive staining of Annexin V-FITC for PS (**left** panel) and A549 EVs binding to 16HBE14o cells (**right** panel); nucleus was stained with DAPI (blue) and images were taken with an SP8 microscope at 100× magnification under oil immersion of EVs alone and at 40× magnification for cells with EVs. (**E**) Representative dot-plots of the flow cytometric analysis of PS exposure. Five micrograms of EVs enriched from the supernatant of 16HBE14o cells (**top** row) and A549 cells (**bottom** row) was stained with Annexin V-V450 in the presence of calcium (**right** column) to detect PS surface exposure. Unstained samples (**left** column) were analyzed to control for eventual sample autofluorescence. Staining in the absence of calcium (**middle** column) was performed to control for specific Annexin V binding.

Isolation of medium EVs was performed as reported [23] and is described in Figure 1A. Determining the size of EVs is critical, prior to performing any downstream analysis. For this, we performed dynamic light scattering (DLS) on both A549- and 16HBE14o-derived EVs and found that both EVs are in the size range of medium EVs (>200–1000 nm). Size distribution of A549 EVs was measured in triplicate and the Z Average was 392.2 nm, whereas the 16HBE14o EVs’ Z average was 300 nm. In addition, data show that isolated EVs are pure, with no peaks in the lower or higher range of medium EVs, and the polydispersity index was around 0.5 and 0.4, indicating favorable polydispersity of EVs. After analyzing size, we characterized the EVs by the commonly known marker tetraspanin CD63 [24]. We observed that CD63 was detected in A549 EVs but not in the cells, which further confirms that we isolated bona fide EVs (Figure 1C). EVs are made up of a lipid membrane, so characterizing the membrane lipids is another way to confirm EVs. We performed Annexin V-FITC staining of phosphatidylserine (PS) on EVs and visualized the EVs by microscopy (Figure 1D). Annexin V binding to PS is reversible and calcium-dependent. We therefore assessed Annexin V binding to A549 and 16HBE14o EVs by flow cytometry in the presence and absence of calcium and confirmed that the fluorescent signal is abrogated in the absence of calcium.

### 2.2. Profiling A549 EVs for Oncogenic Protein Expression and Effects on Cell Migration, Invasion, and Proliferation

**Figure 2:**
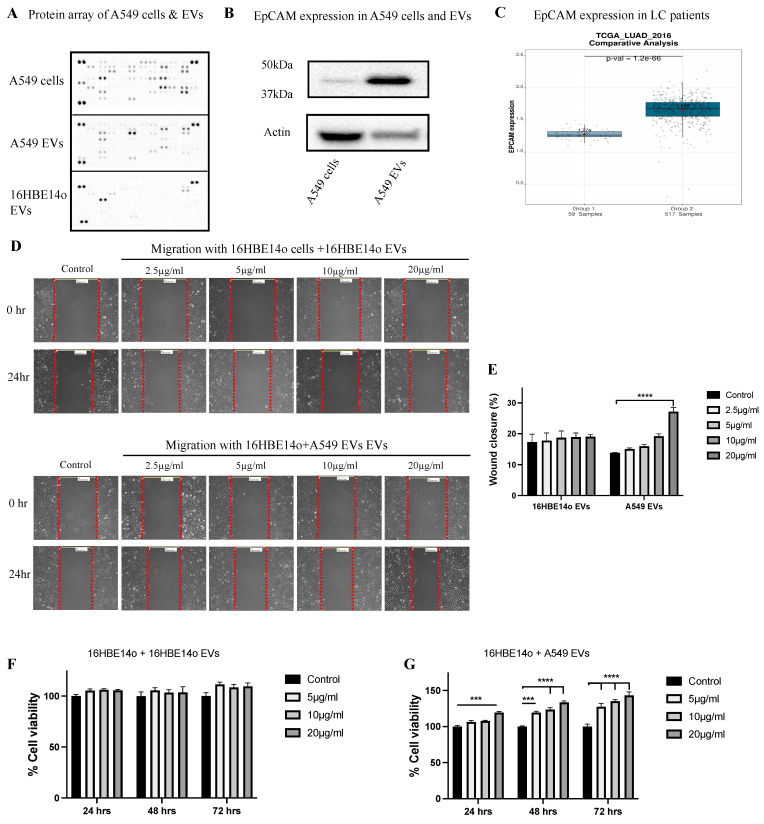

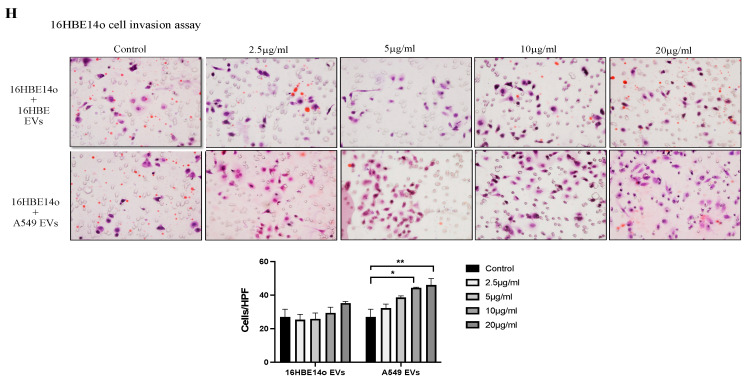
A549 EVs induced tumorigenesis in healthy 16HBE14o cells. (**A**) Human oncology array of A549 cell, A549 EV, and healthy and C-EVs recipient cell protein expression. (**B**) EpCAM protein expression (Western blot) in A549 cells and EVs showing EpCAM is highly encapsulated in A549 EVs. (**C**) EpCAM gene expression analyzed from publicly available LC database. (**D**) The 16HBE14o cell migration included by A549 EVs. Images were taken at 10× magnification. (**E**) The distance between the two edges of scratch was measured at 0 and 24 h. Data were analyzed using a one-way ANOVA: **** *p* < 0.001 vs. control. (**F**,**G**) Proliferation activity of A549EVs by MTT cell viability assay. Data analyzed using a one-way ANOVA: *** *p* < 0.00, **** *p* ≤ 0.0001 vs. control. (**H**) Invasion activity of A549 and 16HBE14o EVs was measured using Boyden’s chamber assay with 16HBE14o cells after 24 h. It was found that 10 and 20 µg/mL of A549 EVs showed invasion compared to control. Values are expressed as mean ± SEM (n = 3 independent experiments) and statistical differences were assessed by one-way ANOVA: * *p* ≤ 0.05, ** *p* ≤ 0.01.

Profiling of EVs is crucial before performing any downstream functional analysis. We conducted an oncology protein array to measure 84 major cancer-related proteins in both A549 cells and A549 EVs and found that EpCAM, EGFR, Dkk-1, Galectine-3, Endostatin, E-Cadherin, FGF basic, Vimentin, and progranulin are the top nine hits (Figure 2A). EpCAM is highly expressed in EVs relative to their parental cells, a finding that we further validated by WB (Figure 2B). We examined the expression of EpCAM in publicly available lung cancer data and found that EpCAM is highly upregulated in LC (https://lce.biohpc.swmed.edu/, accessed on 2 July 2022).

We then assessed the effects of EVs on the migration capacity of 16HBE14o cells. Treatment of 16HBE14o cells with 20 µg/mL protein-equivalent A549 EVs significantly increased cell migration (Figure 2E), whereas no significant change in migration was observed when 16HBE14o cells were treated with 16HBE14o EVs. Cell proliferation assays were performed on 16HBE14o cells treated with A549 EVs and 16HBE14o EVs over 0, 24, 48, and 72 h (Figure 2F,G), and it was found that A549 EVs significantly increased proliferation at 5, 10, and 20 µg/mL of protein equivalent EVs in a dose-dependent fashion. No significant change in proliferation was observed when cells were treated with 16HBE14o EVs. Similar results were observed in an invasion assay with 10 and 20 µg/mL A4549 EVs. These functional assays clearly indicate that C-EVs induced tumorigenesis in healthy cells.

### 2.3. Understanding the Mechanism of A549-EV-Induced Tumorigenesis

**Figure 3:**
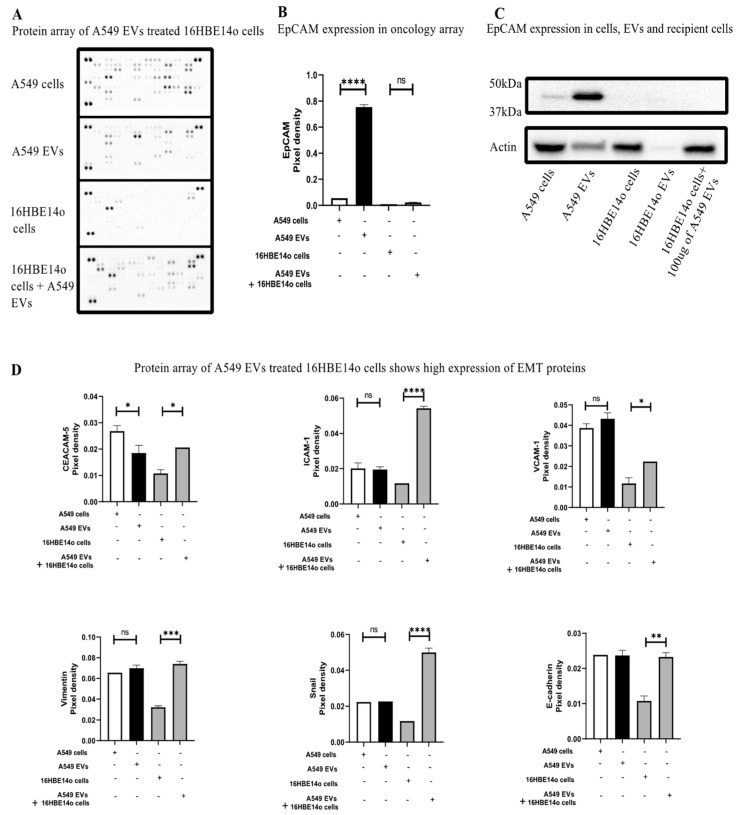
A549 EVs induced tumorigenesis in healthy cells by upregulating EMT markers (**A**) An oncology array of cancer markers upregulated in A549 cells and EVs, 16HBE14o cells and A549 EVs treated 16HBE14o cells shows a high amount of cancer markers was observed on the treated cells. (**B**) EpCAM, which is highly expressed in EVs has been utilized by the healthy cells, as observed by measuring the pixel density of EpCAM among the groups by Image J Fiji (version 1.53). Statistical analysis was done using a One-way ANOVA *** *p* ≤ 0.0001. (**C**) EpCAM expression in the recipient cells was further confirmed by western blot. (**D**) An oncology array shows a significant upregulation of various EMT markers on the recipient cells like CEACAM5, ICAM1, VCAM1, Vimentin, Snail1, and E-cadherin. Pixel density was measured using Image J Fiji and significant differences were evaluated using a One-Way ANOVA ns = non-significant, * *p* > 0.05, ** *p* ≤ 0.05, *** *p* ≤ 0.01, **** *p* <0.001.

To further investigate the mechanism, it is clearly essential to validate the tumorigenesis potential of A549 EVs to induce migration, proliferation, and invasion. We again performed an oncology array on the recipient cells (16HBE14o) (Figure 3A). Our analysis revealed that EpCAM is highly enriched in A549 EVs, and when we treat 16HBE14o cells with A549 EVs, EpCAM can be utilized by the recipient cells (Figure 3B), which was further confirmed by Western blot analysis (Figure 3C). Moreover, the oncology array of the 16HBE14o cells indicated a significant rise of other oncogenic cell adhesion molecules, such as carcinoembryonic antigen-related cell adhesion molecule5 (CEACAM-5), intercellular adhesion molecule-1 (ICAM-1) [25], vascular cell adhesion molecule 1 (VCAM-1), E-Cadherin (epithelial marker) [26], Snail [27], and Vimentin (mesenchymal marker) [28]. Thus, recipient cells exposed to A549 EVs have higher expression of EMT markers, which may promote tumorigenesis.

### 2.4. Understating the Location, Encapsulation, and Uptake of EpCAM 

**Figure 4:**
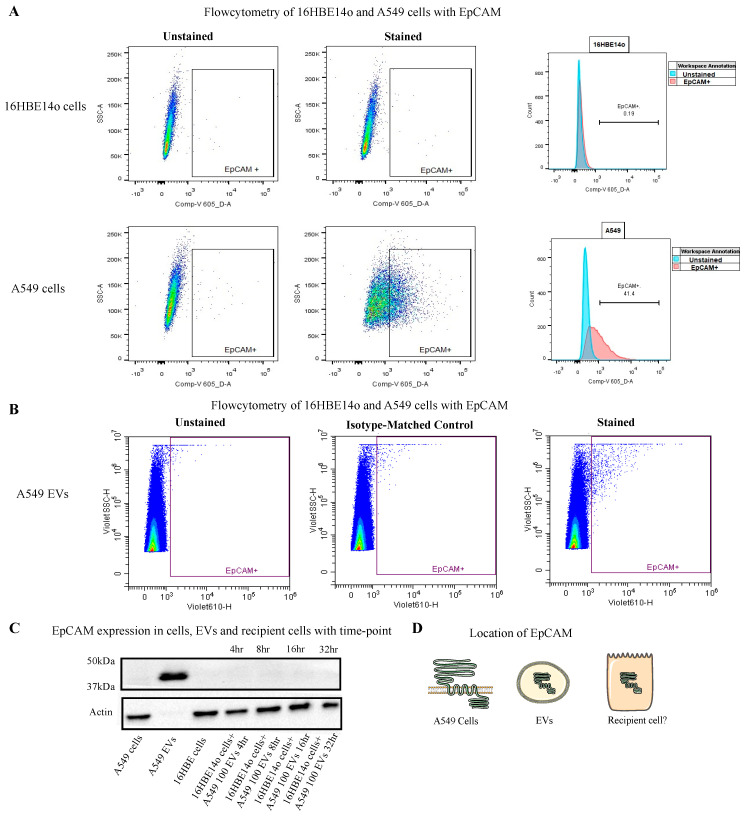
(**A**) To understand the localisation of EpCAM, flow cytometric analysis was performed on 16HBE14o (**upper** row) and A549 cells (**lower** row): 41.4 % of A449 cells stain positive for EpCAM at the surface. (**B**) Flow cytometric analysis of A549 EVs with EpCAM indicating that EpCAM was encapsulated inside the EVs. (**C**) Immunoblotting was performed with EpCAM on the recipient cells at 4, 8, 16, and 32 h timepoints. (**D**) Schematic overview of EpCAM location and utilisation.

Initially, we performed flow cytometric analysis of EpCAM on the surface of healthy 16HBE14o and A549 cells and detected EpCAM on the A549 cells, which indicates that during membrane shredding EpCAM was encapsulated in the EVs (Figure 4A). Flow cytometric analysis of A549 EVs could not detect EVs on the surface, which suggests EpCAM was internalized by the EVs (Figure 4B). Further Western blot analysis of the recipient cells after treatment with A549 EVs at different timepoints (4, 8, 16, and 32 h) showed that, surprisingly, EpCAM seems to be most detected in the recipient cells before 4 h (Figure 4C). Overall, the EpCAM location indicates it is a transmembrane protein on A549 cells, and that it has been encapsulated by A549 EVs as well as been transferred by healthy 16HBE14o cells (Figure 4D).

## 3. Materials and Methods

### 3.1. Cell Culture

Lung adenocarcinoma cell line A549 purchased from ATCC, USA was obtained as a kind gift from Woolcock Institute of Medical Research, Sydney. A549 cells were grown in a Roswell Park Memorial Institute (RPMI) media (Sigma Aldrich, St. Louis, MI, USA) containing 5% heat-inactivated fetal bovine serum (FBS) (Novogen, North Mackay, Australia), 1% penicillin, and streptomycin (Gibco, Waltham, MA, USA) and were maintained in a humidified atmosphere of 5% CO_2_ at 37 °C. All EVs collected from A549 cells were from A549 passage number 20–25 for p consistency. In addition, 16HBE14o-human normal (healthy) bronchial epithelial cells (passage 10–15) were provided by Ling Bi from Charles Perkins Centre—The University of Sydney, Australia, and grown in DMEM supplemented with 10% heat-inactivated FBS and 100 IU/mL penicillin and 100 μg/mL streptomycin in a humidified atmosphere of 5% CO_2_ at 37 °C. Cells were constantly checked for mycoplasma contamination and all experiments were conducted in mycoplasma-negative cells. FBS may contain exosomes that may result in false-positive results during characterization, therefore we performed all experiments in exosome-depleted media. For this, FBS was spun using a Beckman Coulter ultracentrifuge at 100,000× *g* for 18 h [29]. The supernatant was collected, leaving the exosome pellet in the bottom of the tube. The collected FBS was used to prepare 5% or 10% FBS media, referred to as exosome-depleted media [29].

### 3.2. Collection and Isolation of EVs 

A density of 10 × 10^6^ A549 cells were seeded in 12 T-175 flasks. The following day, media were discarded before washing twice with PBS and media were replenished with fresh exosome-depleted media. After 72 h, the supernatant was collected and centrifuged at 500× *g* for 5 min to remove any dead cells or cell debris, then supernatant was centrifuged again at high speed (18,000× *g*) for 90 min at 4 °C, and the pellet-containing EVs were resuspended and washed in serum-free media and further centrifuged for 2 min at 2000× *g* to remove any debris. Finally, the supernatant was further centrifuged for 30 min at 18,000× *g* and 4 °C and the final pellet containing EVs was resuspended in 1 mL PBS and stored at −80 °C for downstream analysis [23]. The 16HBE14o cells were seeded at a density of 10 × 10^6^ into six T-175 flasks. The following day, media were discarded followed by washing twice with PBS, and media were replenished with fresh exosome-depleted media. As healthy cells do not release the same amount of EVs as cancer cells do, we had to stimulate the 16HBE14o cells with lipopolysaccharides (LPS; 1 µg/mL) obtained from *Pseudomonas aeruginosa*. Stimulating healthy cells with LPS to induce the release of significant amounts of EVs is a well-established protocol referred to by many studies [30]. After 48 h of stimulation, media were collected and centrifuged similarly to A549 EVs.

### 3.3. Particle Size Distribution by Dynamic Light Scattering

A small volume of the EV fraction was obtained from A549 and 16HBE14o EVs and further diluted in PBS. Particle size and zeta potential were measured using Zetasizer Nano ZS equipped with He-Ne 633 nm laser light source and a reading was measured at 25 °C in a size range of 0.3–10,000 nm diameter size.

### 3.4. Protein Extraction from EVs, Whole Cell Lysate, and Protein Quantification

RIPA buffer containing phosphatase and protease inhibitor cocktail (Roche, USA) was added to the EV pellet followed by vortex and incubation on ice for 15 min. The EVs were then sonicated at 30% amplitude three times for 2 s, before being incubated again on ice for an additional 15 min. Then, the lysate was then centrifuged for 30 min at 18,000× *g* and 4 °C, before the supernatant was collected, and the protein concentration quantified using a Pierce^TM^ BCA Protein Array Kit (Thermo fisher Scientific, Waltham, MA, USA).

A549 and 16HBE14o cells grown in 6-well plates were allowed to reach approximately 80% confluence before the media were removed, and the cells washed with ice-cold PBS and RIPA lysis buffer containing phosphatase inhibitor cocktail were added. Using a cell scraper, cells were scraped, and the lysate transferred to 1.5 mL tubes followed by incubation on ice for 15 min. The lysate was then sonicated at 30% amplitude three times for 2 s followed by incubation on ice for another 15 min. Finally, the lysate was centrifuged at 12,000× *g* for 15 min at 4 °C, before the supernatant was collected, and protein concentration quantified using a Pierce^TM^ BCA Protein Array Kit.

### 3.5. Western Blotting

Equal amounts of extracted protein were loaded for SDS-PAGE and transferred to PVDF membrane for immunoblotting. After an overnight incubation with primary antibody (Anti-CD63 (ab193349) and Anti-EpCAM (epithelial cell adhesion molecule) (ab223582) at 1:1000 and 1:1500 dilution, respectively, at 4 °C and anti-mouse secondary IgG antibody for CD63 and Anti-rabbit IgG for EpCAM), images were taken using a chemiDoc imaging system. The blots were then stripped with stripping buffer and blocked with 1% BSA, incubated with beta Actin (ab8226) at a 1:10,000 dilution for 2 h at room temperature, washed 3 times with TBST buffer followed by an incubation with anti-mouse secondary antibody (1:10,000 dilutions), and imaged using a chemiDoc.

### 3.6. Immunofluorescence

Freshly isolated EVs were seeded onto collagen-coated slides and stained with Annexin V-FITC (BD Bioscience) for 2 h along with Annexin V binding buffer and images were taken by confocal microscopy (SP8). Images were analyzed using ImageJ. 1 × 10^5^. Then, 16HBE14o cells were seeded onto collagen-coated glass slides and allowed to attach for 6 h. A549 EVs were stained with Annexin V-FITC and seeded onto 16HBE14o cells and allowed to attach for 4 h. ProLong™ Gold antifade mountant was then used for staining the nucleus and microscopic images were taken by confocal microscopy at 40× magnification. 

### 3.7. Flow Cytometry

An amount of 5 µg of A549 and 16HBE14o EVs was analyzed by flow cytometry. Both EVs were stained with V450 Annexin V (BD Horizon^TM^ catalog No. 560506) for 30 min in the dark. Unstained samples were used as a negative control. Initially, 5 µg of unstained samples were run to determine the concentration of EVs for flow cytometry based on the gating strategy. EVs stained with Annexin V were run in the presence or absence of calcium, as Annexin V binding can be determined only in the presence of calcium. Unstained A549 cells and EVs were used as negative controls to establish the gating strategy. A549 EVs and cells stained for EpCAM were then run and plotted for EpCAM binding.

### 3.8. Protein Array of EVs and Cells

Protein from A549 cells, A549 EVs, 16HBE14o cells, and 16HBE14o cells treated with A549 EVs were isolated as previously mentioned, and equal amounts (600 µg) of protein were loaded to perform an oncology array (R&D Systems, Minneapolis, MN, USA) following the manufacturer’s protocol (https://www.rndsystems.com, accessed on 10 January 2022). Data were analyzed using Image J by measuring the pixel density.

### 3.9. Migration (Scratch Wound Healing Assay)

The 16HBE14o cells were plated in a six-well plate at a density of 2 × 10^4^ cells per well. The following day, cells were washed twice with PBS. A vertical straight scratch at the center of the plate was made with a sterile 200 µL pipette tip followed by washing at least 3 times to remove unattached cells. Then, 2 mL of cell culture media was added and images of each well at three different positions (up, middle, and down) were taken at 10× magnification. Baseline images were taken at T = 0 before EVs from A549 cells were treated with increasing protein concentrations of 1 µg/mL, 2.5 µg/mL, 5 µg/mL, 10 µg/mL, and 20 µg/mL. The plates were then incubated at 37 °C for 24 h or 48 h. Images of all wells were then taken again at 10× magnification.

### 3.10. MTT Cell Proliferation Assay

In a 96-well plate, 16HBE14o cells were seeded at a density of 2500 cells per well for 72 h, 5000 cells for 48 h, and 10,000 cells for 24 h. The following day, the media were discarded and any unadhered cells were removed by washing with PBS. Cells were treated with or without proliferation inducers at different concentrations and incubated for 24 h, 48 h, and 72 h depending on the experimental time course. MTT solution was added at 0.5 mg/mL in 100 µL of culture media per well and incubated for 4 h. Media were then discarded and 100 µL of DMSO was added to dissolve the formazan. The absorbance was then measured at 562 nm using a microplate reader (BMG Labtech, Carbine Way, Australia).

### 3.11. Cell Invasion Assay

To determine the effect of EVs on the invasion capacity of 16HBE14o cells, we performed a modified Boyden’s chamber assay using EVs derived from either healthy or cancer cells. We used 24 transwell permeable supports (6.5 mm insert, 8 μM pore size polycarbonate membrane), precoated on the lower surface of the membranes with 2.5% gelatin in 1 M acetic acid for 1 h. The 16HBE14o cells were seeded in the upper chamber (200 cells/well in 200 μL of DMEM), and inserts were then placed in a well containing 600 μL of DMEM. Cells were allowed to adhere and then treated with A549- or 16HBE14o-derived EVs (0, 2.5, 5, 10, or 20 µg/mL) and incubated for 24 h. The cells from the upper transwell surface were removed by cotton swabs, and the membrane was cut and fixed in 10% formalin and stained with hematoxylin and eosin. Cells that had crossed the pores of the membrane to the lower surface were counted in 5 random fields (magnification 20×), and cell invasion was calculated as average cells per field of view [31].

### 3.12. Statistical Analysis

The values are represented as mean ± SEM. Graph Pad Prism (version 9.3) was used to perform statistical analyses. Statistical comparisons were conducted by unpaired, two-tailed Student’s *t* test for two groups and one-way ANOVA for more than two groups. A value of *p* < 0.05 was considered statistically significant.

## 4. Discussion

EVs have the capacity to alter the phenotype of recipient cells from healthy to malignant when internalized by the healthy cell. This is primarily mediated by the transfer of oncogenic proteins and RNA present in cancer-cell-derived EVs [8,32]. However, it remains unknown how exactly they are internalized and how they develop a cancer-related phenotype in terms of the mechanistic and molecular regulation of pathways in the recipient cell. This study provides further understanding of how C-EVs induce a tumor-like phenotype in healthy bronchial epithelial cells, mostly at the protein level. 

We initially focused on the isolation of A549 and 16HBE14o medium-sized EVs by separating them from sEVs and large oncosomes, which is critical before any further downstream analysis can be conducted [33]. According to the *Minimal information for studies of extracellular vesicles 2018 (MISEV2018)* guidelines [1], isolated EVs should be characterized by at least two well-established methods [26]. In our study, we initially characterized EVs by DLS, confirming a size consistent with medium EVs, then verified protein expression of tetraspanin CD63 as an EV marker using Western blot. Furthermore, we performed flow cytometric analysis and microscopy for phosphatidylserine surface staining using Annexin V (Figure 1) [29]. 

We analyzed the oncogenic proteins which are encapsulated in A549 EVs and found that EpCAM, EGFR, Dkk-1, Galectine-3, Endostatin, E-Cadherin, FGF basic, Vimentin, and progranulin were the top nine hits on the oncology blot. Interestingly, all these proteins are connected to the WNT/β-catenin signaling pathway. Among them, DKK1 and Endostatin are downregulators of this pathway [34,35,36,37,38,39,40,41,42]. To see the effect of these oncogenic proteins within the EVs, we treated healthy bronchial epithelial cells with A549 EVs and elevated changes in the oncogenic activity of the healthy cells by using assays for cell migration, invasion, and proliferation. Interestingly, we found a significant increase in the cell proliferation, migration, and invasion activity in the recipient healthy cells (16HBE14o) (Figure 2).

**Figure 5:**
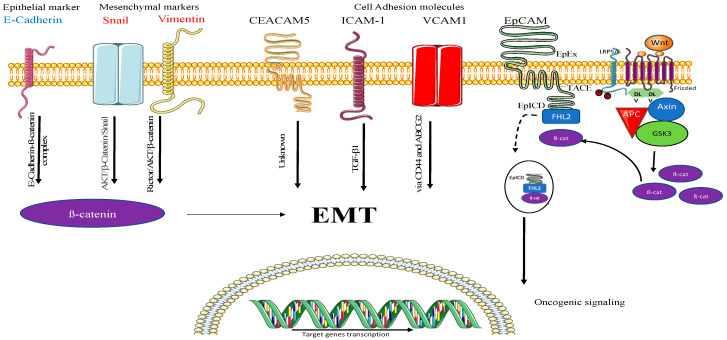
Predicted mechanism of tumorigenesis. (From **left**) Epithelial marker E-Cadherin was found on recipient cells, which is known to form a complex with β-catenin and can thereby promote EMT. Mesenchymal markers such as Snail and Vimentin further support our hypothesis, which are known to interact with β-catenin via AKT/β-Catenin/Snail and Rictor/AKT/β-catenin complexes, respectively. Cell adhesion molecules are surface proteins involved in cell–cell communication, and CEACAM-5 is known to induce tumorigenesis, but its exact role is still unknown in terms of EMT. ICAM-1 is known to induce EMT by regulating TGF-β1. Another cell adhesion molecule, VCAM-1, is known to induce EMT via CD44 and ABCG2 expression. Finally, EpCAM is taken up by the recipient cells, utilized by breaking down of EpCAM by tumor necrosis factor α-converting enzyme (TACE), which is known as EpICD, which merges with four-and-a-half LIM domain protein 2 (FHL2) forming a complex with β-catenin and further activates oncogenic signaling.

Furthermore, we performed an oncology array on the EV-treated recipient cells and found that the highly expressed protein on A549 EVs, EpCAM, was efficiently transferred to healthy cells [43], a finding that we further confirmed by WB (Figure 3). The use of EpCAM in recipient cells is already demonstrated in the literature, where EpCAM is cleaved by β-catenin, leading to activation of EMT and further leading to tumorigenesis [44,45]. In contrast, we saw a significant increase in other oncogenic cell adhesion molecules including CEACAM-5, ICAM-1 [25], and VCAM-1 [46], epithelial markers such as E-Cadherin [26], as well as mesenchymal markers such as Snail [27] and Vimentin [28] in the recipient cells. Interestingly, all these markers are part of the EMT and mainly regulated by β-catenin signaling, but our findings suggest for the first time that C-EVs can activate EMT markers in healthy cells, leading to tumorigenesis [47,48,49,50,51,52,53,54] (Figure 5). Overall, the use of a β-catenin pathway inhibitor could be a potential therapy for C-EV-induced tumorigenesis/metastasis, and detection of these EV markers in biological fluids can be a potential diagnostic application.

## 5. Conclusions

Overall, we have shown how different EMT signaling proteins induce tumorigenic changes in a healthy cell, namely by increasing cell migration, proliferation, and invasive potential. In addition, the highly expressed protein EpCAM was completely taken up by healthy cells, as they could not be detected by an oncology array or Western blot, indicating some proteins are directly utilized by cells following their uptake. Interestingly, many EMT and cell adhesion molecule related proteins were detected on the recipient cells (Figure 5), which suggests they are enhancing tumorigenesis by regulating β-catenin and eventually EMT. Thus, this study gives further evidence that cancer cell EVs carry oncogenic proteins which are actively taken up by healthy cells to induce tumorigenesis. Finally, this study helps in better understanding EMT induced by C-EVs. The validation of these findings with an inhibitor could be a potential therapy for LC. 

## Data Availability

The data presented in this study are available in the manuscript.
